# Screening of Promising Chemotherapeutic Candidates from Plants against Human Adult T-Cell Leukemia/Lymphoma (VII): Active Principles from *Thuja occidentalis* L.

**DOI:** 10.3390/molecules26247619

**Published:** 2021-12-15

**Authors:** Daisuke Nakano, Kenji Ishitsuka, Madoka Ishihara, Ryota Tsuchihashi, Masafumi Okawa, Kazuo Tamura, Junei Kinjo

**Affiliations:** 1 Faculty of Pharmaceutical Sciences, Fukuoka University, Fukuoka 814-0180, Japan; dnakano@fukuoka-u.ac.jp (D.N.); pp110077fu@gmail.com (M.I.); ryouta-t@fukuoka-u.ac.jp (R.T.); kinjojun@fukuoka-u.ac.jp (J.K.); 2 Division of Hematology and Rheumatology, Graduate School of Medical and Dental Sciences, Kagoshima University, Kagoshima 890-8544, Japan; kenji-i@m.kufm.kagoshima-u.ac.jp; 3 Division of Medical Oncology, Hematology and Infectious Disease, Department of Internal Medicine, Fukuoka University, Fukuoka 814-0180, Japan; ktamura@fukuoka-u.ac.jp

**Keywords:** screening, adult T-cell leukemia/lymphoma, *Thuja occidentalis*

## Abstract

During the screening of novel chemotherapeutic candidates from plants against adult T-cell leukemia/lymphoma, we identified that the extracts of *Thuja occidentalis* (Cupressaceae) showed potent anti-proliferative activity in MT-1 and MT-2 cells. Therefore, we attempted to isolate the active components from this plant. We isolated and identified 32 compounds (**1**–**32**; eight lignans, 18 terpenoids, and six flavonoids) from the extracts of the leaves and cones. Their structures were determined by spectroscopic analysis. Several of the isolated compounds inhibited the growth of both cell lines. Lignans showed more potent activity than other classes of compounds. A comparison of the activities of compounds **1**–**8** revealed that the presence of a trans-lactone (linkage of C-6 to C-7) correlated with increased activity. Diterpenes showed moderate activity, and the presence of a ketone moiety at the C-7 position correlated with increased activity in compounds **12**–**21**. In addition, biflavones showed moderate activity, and the presence of methoxy functions appeared to influence the activity of these compounds. Several lignans were lead compound of anti-cancer reagent (etoposide). In conclusion, not only lignans, but also diterpenes and/or biflavones, may be promising candidates for the treatment of adult T-cell leukemia/lymphoma.

## 1. Introduction

Adult T-cell leukemia/lymphoma (ATL) is a malignancy of mature peripheral T-lymphocytes associated with human T-cell lymphotropic virus type I (HTLV-1). Conventional chemotherapeutic regimens used to treat other types of malignant lymphoma have been administered to ATL patients, but the therapeutic outcomes of acute- and lymphoma-type ATL remain very poor [1]. Therefore, we conducted a search for novel chemotherapeutic candidates for the treatment of ATL in plant extracts and tested these compounds in two cell lines. MT-1 cells were established from the peripheral blood tumor cells of ATL patients [2], and MT-2 cells were established from cord blood T-cells by co-cultivation of normal human cord lymphocytes and peripheral blood tumor cells from an ATL patient [3].

We previously reported the screening of 582 extracts and the isolation of active constituents (withanolides, cardenolides, aporphine alkaloids, phenanthroindolizidine alkaloids, quinolone alkaloids, and coumarins) for testing in MT-1 and MT-2 cell lines [4,5,6,7,8,9,10]. In a previous paper, induction of apoptosis by 24,25-dihydrowithanolide D determined by cytometric APO2.7-PC5 assay and evidenced by PARP cleavage [6], calotropin (cardenolide) induced apoptosis in MT-1 and MT-2 cells in a concentration-dependent manner, and the cell cycle experiments demonstrated that calotropin arrested MT-1 and MT-2 cells at the G2/M phase [7], accumulation of Sub-G1 cells were observed in MT-1 and MT-2 cells treated by liriodenine (aporphine alkaloid), suggesting induction of apoptosis [8]. Herein, following the results of our previous screening program [5], we report the isolation of active compounds from *Thuja occidentalis*, a plant belonging to the family Cupressaceae. *T. occidentalis* is native to North America and widely cultivated as ornamental tree known as white cedar [11]. Previous phytochemical studies on *T. occidentalis* have resulted in the isolation of several diterpenes (dehydroabietane, neothujic acids III and IV), lignans [(−)-matairesinol, (−)-thujaplicatin methyl ether, (−)-wikstromol, *epi*-pinoresinol], monoterpenes (α-thujone, β-thujone, fenchone), and a sesquiterpene alcohol [(+)-occidentalol] [12]. In traditional medicine, *T. occidentalis* has been used in the treatment of liver diseases, bronchitis, psoriasis, enuresis, amenorrhea, cystitis, uterine carcinomas, diarrhea, and rheumatism [13]. Thujone exerts proapoptotic and antiinvasive effects on GBM cells. They confirm the potential of α-thujone for the treatment of glioblastoma multiforme [14]. In in vivo studies, α/β-thujone promotes the regression of neoplasia and inhibited the angiogenic markers (VEGF, Ang-4, and CD31) into the tumor [15]. The aqueous extract and the polysaccharide fraction of *T. occidentalis* reduced production of proinflammatory cytokines (TNF-α and IL-6), decreased immunostaining of COX-2 and iNOS, and inhibited oxidative stress [16].

## 2. Results and Discussion

### 2.1. Screening of Antiproliferative Activities of Cupressaceae Plants against MT-1 and MT-2 Cells

Table 1 summarizes the anti-proliferative activities of several Cupressaceae plant extracts based on their activity in MT-1 and MT-2 cells. Despite variation in potency, anti-proliferative activity was exhibited by nine of the 10 extracts. In a preliminary test, the most potent anti-proliferative activity in MT-1 and MT-2 cells was exhibited by leaf extract following by the cone extract of *T. occidentalis*; therefore, we attempted to isolate the active compounds from these extracts. In addition, we attempted to isolate an extract from the cones of *T. occidentalis*.

### 2.2. Isolation of Compounds from the Extracts of T. occidentalis

Extraction of the leaves of *T. occidentalis* was performed with MeOH under reflux, and the extract was initially partitioned between *n*-hexane and 80% MeOH. The *n*-hexane layer and 80% MeOH layer were subjected to columns to isolate the compounds. Conversely, extraction of the cones of *T. occidentalis* was performed with MeOH under reflux and the extracts were subjected to columns to isolate the compounds.

**Figure 1:**
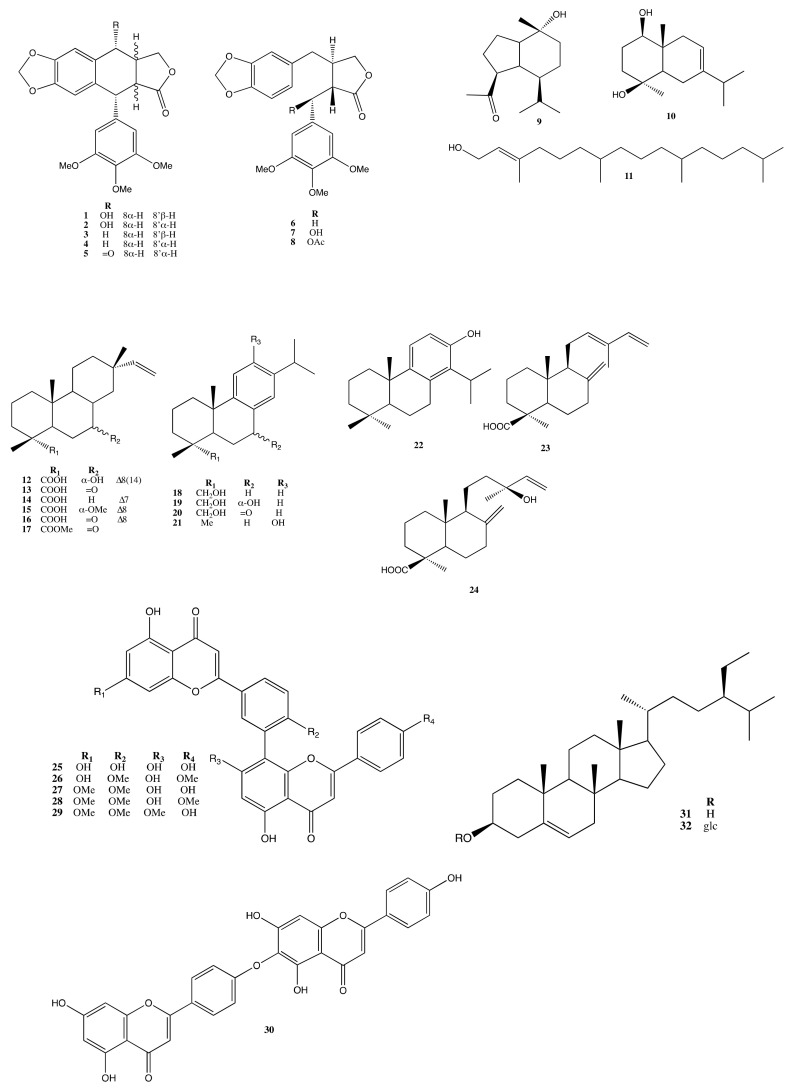
Compounds from *T. occidentalis*.

The extracts were subjected to several chromatographic purification steps to obtain compounds **3**–**9**, **11**–**18**, **20**–**27**, and **29**–**32** from the leaves; compounds **1**–**3**, **6**, **9**–**10**, **19**–**20**, and **27**–**29** were extracted from the cones (Figure 1). Compounds **1**–**32** had been previously identified and the physical data of these compounds are in accordance to those reported [17,18,19,20,21,22,23,24,25,26,27,28,29,30,31,32,33,34,35,36,37,38,39,40].

### 2.3. Determination of Anti-Proliferative Activity

The anti-proliferative effects of compounds **1**–**32** are listed in Table 2. In addition, doxorubicin (clinically used antineoplastic drug) and etoposide (semisynthetic derivative of podophyllotoxin) were used as positive control.

**Figure 2:**
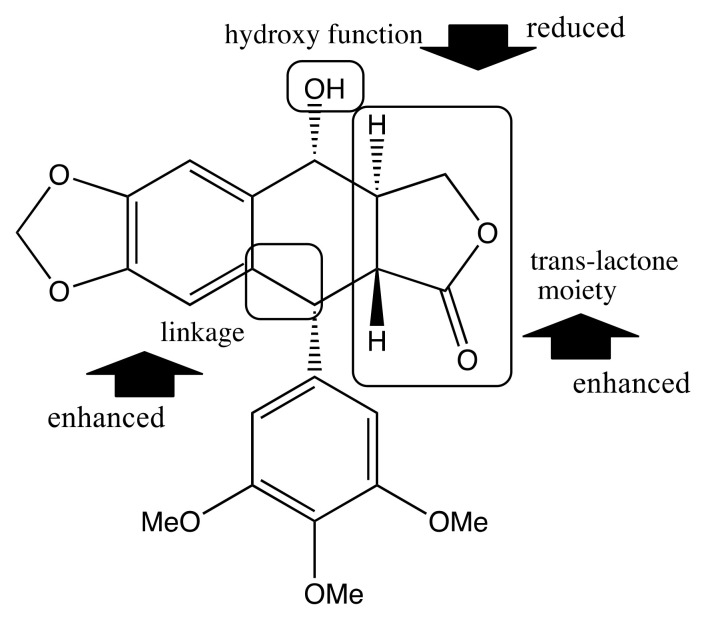
Structure–activity relationships of lignans.

Almost all of the isolated lignans (**1**–**8**) inhibited the growth of both tumor cell lines. But all compounds had weaker antiproliferative activity than positive control except compound **3**. The trans-lactone moiety in compounds **1** and **3** increased their anti-proliferative activity compared with compounds **2** and **4**, which do not possess a trans-lactone moiety. The presence of a hydroxyl group at position 7α in compounds **1** and **2** appeared to decrease the activity of these compounds compared with compounds **3** and **4**. Furthermore, the presence of a ketone moiety at position C-7 in compound **5** was associated with decreased activity in comparison to compound **4**, which lacked this moiety. Comparison of compounds **3** and **6** indicated that, the linkage of C-6 to C-7 appeared to enhance the activity (Figure 2).

Compared with compounds **6**, **7**, and **8**, the presence of substituents (OH and OAc) influenced the activities.

**Figure 3:**
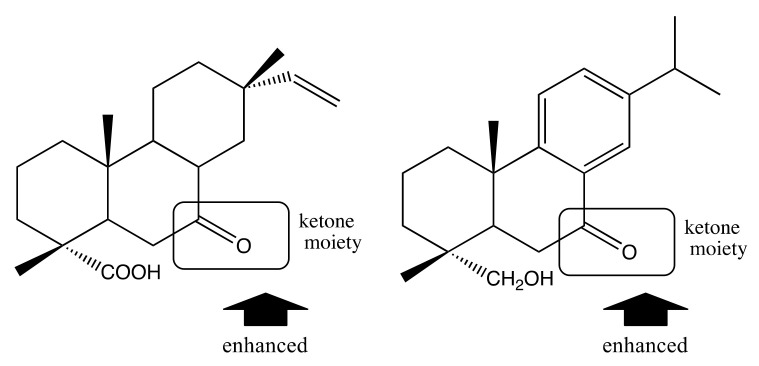
Structure–activity relationships of diterpenoids.

Several of the isolated terpenoid compounds inhibited the proliferation of cell lines. The presence of a double bond at position C-8 in compound **16** appeared to enhance its activity compared with compound **13**, which did not have a double bond at this position. Compared with compounds **15** and **16**, the presence of a ketone moiety at position C-7 enhanced the activity. Similarly, compared with compounds **18**, **19**, and **20**, the substituents (OH and ketone) influenced their activities (Figure 3).

Several of the isolated flavonoids (**25**–**30**) inhibited the proliferation of cell lines. Comparison of compounds **25**, **26**, **27**, **28**, and **29**, indicated the presence of one or two methoxy groups appeared to influence the activity of the compounds.

The EC_50_ value of compound **3** against MT-1 and MT-2 cell growth showed that compound **3** had the highest activity compared with the other compounds. Compounds **19** and **20** showed moderate activity in the current study. In previous studies, compound **19** showed cytotoxic activity in LOVO cells [32] and compound **20** showed cytotoxic activity in Hela cells and A549 cells [41]. In addition, both compounds have shown inhibition against Herpes Simplex Virus type 2 (HSV-2) [42]. Compounds **26** and **27** had moderate activity; these biflavones have shown anti-cancer properties in medulloblastoma, leukemia, osteosarcoma, colon, lung, neck, prostate, breast, cervical, ovarian, and kidney cancers [43]. Compound **27** inhibited the growth of Daoy and D283 cell lines, and induced G2/M cell cycle arrest in Daoy cells. Moreover, compound **27** reduced the expression of Wnt target genes, including Axin2 and cyclin D1, and inhibited the survival of MB cells [44].

### 2.4. Apoptosis Analysis

**Figure 4:**
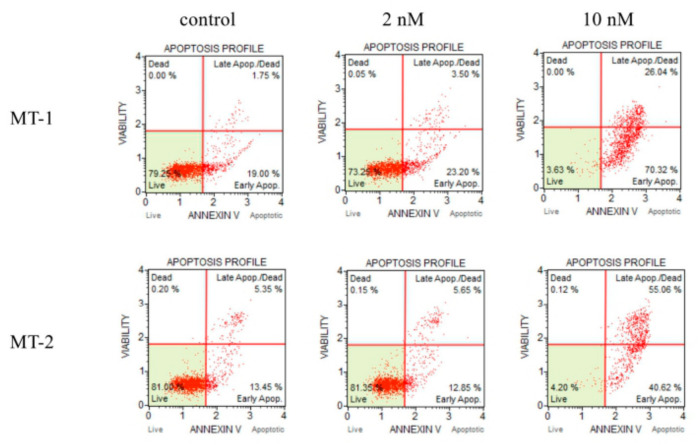
Deoxypodophyllotoxin (**3**) induced cell apoptosis in MT-1 and MT-2 cells.

**Figure 5:**
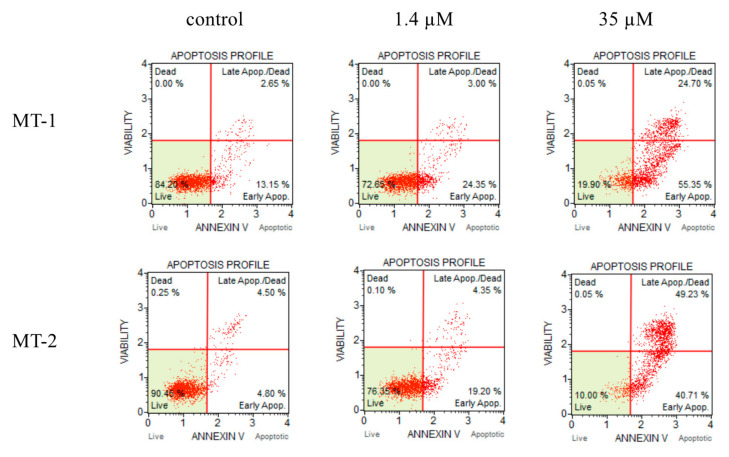
Isoginkgetin (**26**) induced cell apoptosis in MT-1 and MT-2 cells.

We analyzed the ability of the extracted compounds to induce apoptosis. The following experiments were studied for compound **3** which was the most active and compound **26** which was the most active except for lignans. Annexin V-positive cells were used to investigate the effects of deoxypodophyllotoxin (**3**) and isoginkgetin (**26**) on apoptosis. The proportion of annexin V-positive cells after the 72 h treatment with deoxypodophyllotoxin (**3**) and isoginkgetin (**26**) is shown in Figure 4 and Figure 5, respectively. The distribution of apoptotic cells, which were located in the upper-right (late apoptotic/dead cells) and lower-right (early apoptotic cells) quadrants, was increased in cells treated with deoxypodophyllotoxin (**3**) and isoginkgetin (**26**). The concentration of deoxypodophyllotoxin (**3**) was changed 2 nM to 10 nM increased the early apoptotic cells from 23.20% to 70.32%, the concentration of isoginkgetin (**26**) was changed 1.4 µM to 35 µM increased the early apoptotic cells from 24.35% to 55.35%. In the case MT-2 had a similar tendency. Therefore, deoxypodophyllotoxin (**3**) and isoginkgetin (**26**) significantly induced apoptosis in MT-1 and MT-2 cells.

## 3. Materials and Methods

### 3.1. General Experimental Procedures

The ^1^H-NMR (600 MHz) and ^13^C-NMR (150 MHz) spectra were measured in CDCl_3_ or DMSO-*d_6_* using a JNM-ECZ600R spectrometer (JEOL, Tokyo, Japan) at room temperature, and the chemical shifts given as a δ (ppm) scale with tetramethylsilane (TMS) as the internal standard. The FAB-MS was measured using a JEOL JMS-HX110 mass spectrometer and acquired in a glycerol matrix. HPLC was conducted using a Waters machine equipped with a 1525 binary pump and a 2489 UV/Vis detector (Waters, Massachusetts, USA). Separation was carried out using a Cosmosil 5C_18_ MS-II column (20.0 mm × 250 mm, ODS, 5 µm; Nacalai Tesque, Kyoto, Japan). Apoptosis analyses were carried out using a Muse Cell Analyzer (Merck KGaA, Darmstadt, Germany).

### 3.2. Plant Materials

The plant materials used in this study were taken from the medicinal plant garden of Fukuoka University, located in Fukuoka, Japan. Voucher specimens were deposited in the Laboratory of Pharmacognosy of Fukuoka University, Fukuoka, Japan (FUN-0270).

### 3.3. Extraction and Isolation

The samples were powdered, and the compounds were extracted in accordance with the procedure described in a previous paper [4]. Extraction of the leaves of *T. occidentalis* (4860 g) was performed with MeOH under reflux. The extract (72.6 g) was initially partitioned using *n*-hexane and 80% MeOH. The *n*-hexane phase was subjected to a Diaion HP-20 column using 85% MeOH, 90% MeOH, 95% MeOH, 100% MeOH, and MeOH:acetone (4:1) to obtain fraction (fr.) LH1, LH2, LH3, LH4, and LH5, respectively. The fr. LH1 was subjected to a silica gel column using hexane:acetone (9:1) to obtain fr. LH1-1. The fr. LH1-1 was subjected to a silica gel column using hexane:EtOAc (3:1 and 2:1) to obtain fr. LH1-1-1 and compound **13** (1.9 mg). The fr. LH1-1-1 was subjected to HPLC purification using 55% MeCN to obtain compound **17** (22.0 mg). The fr. LH2 was subjected to a silica gel column using hexane:acetone (30:1, 10:1, and 5:1) to obtain fr. LH2-1, LH2-2, and LH2-3. The fr. LH2-1 was subjected to a silica gel column using hexane to obtain fr. LH2-1-1 and LH2-1-2. The fr. LH2-1-1 was subjected to a silica gel column using hexane:acetone (11:1) to obtain fr. LH2-1-1-1. The fr. LH2-1-1-1 was subjected to HPLC purification using 80% MeCN to obtain compound **21** (13.2 mg) and compound **22** (46.4 mg). The fr. LH2-1-2 was subjected to an ODS column using 60% MeOH to obtain compound **14** (517.0 mg). The fr. LH2-2 was subjected to a silica gel column using hexane:acetone (10:1) to obtain fr. LH2-2-1. The fr. LH2-2-1 was subjected to HPLC purification using 90% MeCN to obtain compound **15** (10.9 mg). The fr. LH2-3 was subjected to a silica gel column using hexane:acetone (4:1 and 3:1) to obtain fr. LH2-3-1 and LH2-3-2, respectively. The fr. LH2-3-1 was subjected to a silica gel column using CHCl_3_:MeOH (30:1) to obtain fr. LH2-3-1-1. The fr. LH2-3-1-1 was subjected to HPLC purification using 60% MeCN to obtain compound **20** (20.5 mg). The fr. LH2-3-2 was subjected to a silica gel column using CHCl_3_:MeOH (25:1) to obtain fr. LH2-3-2-1. The fr. LH2-3-2-1 was subjected to HPLC purification using 50% MeCN to obtain compound **16** (18.0 mg). The fr. LH3 was subjected to a silica gel column using hexane:acetone (95:1) to obtain fr. LH3-1. The fr. LH3-1 was subjected to HPLC purification using 80% MeCN to obtain compound **18** (19.8 mg). The fr. LH4 was subjected to a silica gel column using hexane:EtOAC (10:1) and CHCl_3_:MeOH:H_2_O (17:3:0.3) to obtain fr. LH4-1 and LH4-2, respectively. The fr. LH4-1 was subjected to a silica gel column using hexane:acetone (50:1) to obtain fr. LH4-1-1. The fr. LH4-1-1 was subjected to HPLC purification using 85% MeCN to obtain compound **11** (13.6 mg). The fr. LH4-2 was subjected to a silica gel column using acetone to obtain compound **32** (18.0 mg). The fr. LH5 was subjected to a silica gel column using hexane:EtOAc (5:1) to obtain fr. LH5-1. The fr. LH5-1 was subjected to a silica gel column using hexane:acetone (10:1) to obtain fr. LH5-1-1. The fr. LH5-1-1 was subjected to HPLC purification using MeOH to obtain compound **31** (26.3 mg).

The 80% MeOH phase was subjected to a Diaion HP-20 column using MeOH, MeOH:acetone (3:2), and acetone to obtain fr. LM1, LM2, LM3, and LM4. The fr. LM1 was subjected to an MCI gel column using 80% MeOH, 90% MeOH, and acetone to obtain fr. LM1-1, LM1-2, and LM1-3, respectively. The fr. LM1-1 was subjected to a silica gel column using hexane:acetone (5:1) to obtain fr. LM1-1-1. The fr. LM1-1-1 was subjected to a silica gel column using hexane:EtOAc (2:1) to obtain fr. LM1-1-1-1. The fr. LM1-1-1-1 was subjected to a silica gel column using CHCl_3_:MeOH (100:1) to obtain fr. LM1-1-1-1-1. The fr. LM1-1-1-1-1 was subjected to HPLC purification using 40% MeCN to obtain compound **9** (2.5 mg). The fr. LM1-2 was subjected to a silica gel column using hexane:acetone (6:1, 5:1, 3:1, 2:1, and acetone) to obtain fr. LM1-2-1, LM1-2-2, LM1-2-3, LM1-2-4, and LM1-2-5, respectively. The fr. LM1-2-1 was subjected to a silica gel column using CHCl_3_:MeOH (84:1) to obtain compound **24** (6.2 mg). The fr. LM1-2-2 was subjected to a silica gel column using hexane:acetone (13:2) to obtain fr. LM1-2-2-1. The fr. LM1-2-2-1 was subjected to an ODS column using 77% MeOH to obtain compound **12** (22.0 mg). The fr. LM1-2-3 was subjected to a silica gel column using CHCl_3_ and CHCl_3_:MeOH (100:1) to obtain compound **7** (6.7 mg) and fr. LM1-2-3-1, respectively. The fr. LM1-2-3-1 was subjected to HPLC purification using 50% MeCN to obtain compound **8** (5.7 mg). The fr. LM1-2-4 was subjected to a silica gel column using hexane:acetone (2:1) to obtain fr. LM1-2-4-1. The fr. LM1-2-4-1 was subjected to a silica gel column using CHCl_3_ to obtain fr. LM1-2-4-1-1. The fr. LM1-2-4-1-1 was subjected to HPLC purification using 40% MeCN to obtain compound **23** (4.1 mg). The fr. LM1-2-5 was subjected to a silica gel column using CHCl_3_:MeOH:H_2_O (9:1:0.1) to obtain fr. LM1-2-5-1. The fr. LM1-2-5-1 was subjected to an ODS column using 30% MeCN to obtain compound **25** (9.2 mg). The fr. LM1-3 was subjected to HPLC purification using 50% MeCN to obtain compound **27** (45.7 mg) and compound **26** (6.5 mg). The fr. LM2 was subjected to a silica gel column using hexane:EtOAC (3:2) to obtain fr. LM2-1. The fr. LM2-1 was subjected to a silica gel column using hexane:acetone (3:1 and 5:2) to obtain compound **6** (23.9 mg), compound **3** (730 mg), and fr. LM2-1-1. The fr. LM2-1-1 was subjected to HPLC purification using 35% MeCN to obtain compound **4** (2.7 mg) and compound **5** (4.3 mg). The fr. LM3 was subjected to a silica gel column using hexane:EtOAc (1:1) to obtain fr. LM3-1. The fr. LM3-1 was subjected to a silica gel column using CHCl_3_:MeOH (80:1) to obtain fr. LM3-1-1. The fr. LM3-1-1 was subjected to HPLC purification using 50% MeCN to obtain compound **29** (2.2 mg). The fr. LM4 was subjected to a silica gel column using CHCl_3_:MeOH (15:1) to obtain fr. LM4-1. The fr. LM4-1 was subjected to HPLC purification using 45% MeCN to obtain compound **30** (4.6 mg).

Extraction of the cones of *T. occidentalis* (688 g) was performed with MeOH under reflux. The extract (72.6 g) was subjected to a Diaion HP-20 column using 80% MeOH, 100% MeOH, and acetone to obtain fr. C1, C2, and C3, respectively. The fr. C1 was subjected to a silica gel column using CHCl_3_:MeOH:H_2_O (9:1:0.1) to obtain fr. C1-1. The fr. C1-1 was subjected to a silica gel column using hexane:acetone (8:1, 7:1, and 2:1) to obtain fr. C1-1-1, C-1-1-2, and C1-1-3, respectively. The fr. C1-1-1 was subjected to HPLC purification using 50% MeCN to obtain compound **9** (3.2 mg). The fr. C1-1-2 was subjected to a silica gel column using CHCl_3_:MeOH (80:1) to obtain fr. C1-1-2-1. The fr. C1-1-2-1 was subjected to HPLC purification using 50% MeCN to obtain compound **10** (14.0 mg) and compound **19** (3.0 mg). The fr. C1-1-3 was subjected to a silica gel column using hexane:EtOAc (2:3) to obtain fr. C1-1-3-1. The fr. C1-1-3-1 was subjected to HPLC purification using 30% MeCN to obtain compound **2** (2.8 mg). The fr. C2 was subjected to a silica gel column using CHCl_3_:MeOH (20:1) to obtain fr. C2-1. The fr. C2-1 was subjected to a silica gel column using hexane:acetone (8:1, 6:1, and 4:1) to obtain fr. C2-1-1, C2-1-2, and C2-1-3, respectively. The fr. C2-1-1 was subjected to a silica gel column using hexane:EtOAc (7:2) to obtain fr. C2-1-1-1. The fr. C2-1-1-1 was subjected to HPLC purification using 60% MeCN to obtain compound **20** (9.2 mg). The fr. C2-1-2 was subjected to a silica gel column using CHCl_3_:MeOH (40:1) to obtain fr. C2-1-2-1. The fr. C2-1-2-1 was subjected to HPLC purification using 55% MeCN to obtain compound **1** (10.5 mg). The fr. C2-1-3 was subjected to a silica gel column using hexane:EtOAc (2:1) to obtain fr. C2-1-3-1. The fr. C2-1-3-1 was subjected to HPLC purification using 40% MeCN to obtain compound **3** (63.0 mg) and compound **6** (16.2 mg). The fr. C3 was subjected to a silica gel column using hexane:EtOAc (1:1) to obtain fr. C3-1. The fr. C3-1 was subjected to a silica gel column using CHCl_3_:MeOH (25:1) to obtain fr. C3-1-1 and C3-1-2. The fr. C3-1-1 was subjected to HPLC purification using 60% MeCN to obtain compound **29** (3.1 mg) and compound **28** (2.0 mg). The fr. C3-1-2 was subjected to HPLC purification using 45% MeCN to obtain compound **27** (5.3 mg).

### 3.4. Identification of Compounds

Compounds **1**–**32** were identified as podophyllotoxin (**1**) [17], picropodophyllotoxin (**2**) [17], deoxypodophyllotoxin (**3**) [18], deoxypicropodophyllotoxin (**4**) [19], picropodophyllone (**5**) [20], (−)-yatein (**6**) [21], podorhizol (**7**) [22], podorhizol acetate (**8**) [23], oplopanone (**9**) [24], oplodiol (**10**) [25], phytol (**11**) [26], 7α-hydroxysandaracopimaric acid (**12**) [18], 7-oxo-*epi*-pimara-15-en-18-oic acid (**13**) [27,28], isopimaric acid (**14**) [29], macrophypene C (**15**) [30], 7-oxo-*epi*-pimara-8,15-dien-19-oic acid (**16**) [28], 7-oxo-*epi*-pimara-15-ene-18-oic acid methyl ester (**17**) [28], 4-*epi*-dehydroabietinol (**18**) [31], 8,11,13-abietatriene-7α, 18-diol (**19**) [32], 7-oxodehydroabietinol (**20**) [33], ferruginol (**21**) [34], (+)-totarol (**22**) [35], *E*-communic acid (**23**) [18], (+)-cupressic acid (**24**) [36], amentoflavone (**25**) [37], isoginkgetin (**26**) [37], ginkgetin (**27**) [37], 7,4′,4′″-trimethyl-amentoflavone (**28**) [38], 7,4′,7″- trimethyl-amentoflavone (**29**) [38], hinokiflavone (**30**) [37], β-sitosterol (**31**) [39], and β-sitosterol glycoside (**32**) [40], respectively, based on the comparison of their physical data with those reported in the literature (Tables S1–S5).

### 3.5. Cell Culture

Two HTLV-1-infected T-cell lines, MT-1 and MT-2, were kindly provided by Dr Isao Miyoshi of Kochi University in Nankoku, Japan. Culture conditions were as previously described [5]. The cells were cultured in RPMI-1640 medium with *L*-glutamine and sodium bicarbonate containing 15% fetal bovine serum (Biowest, Nuaille, France) and 1% kanamycin. Cells were cultured at 37 °C in humidified 5% CO_2_/95% air.

### 3.6. Measurement of Anti-Proliferative Effects against MT-1 and MT-2 Cells

Viability was determined using the MTT assay. The MT-1 and MT-2 cells were maintained in RPMI-1640 medium containing fetal bovine serum (15%). A 50-μL aliquot of the cell suspension (5000 cells per well) and 50 μL of the test sample solution or suspension were plated in flat-bottomed microtiter wells (extract final concentration: 100, 10, 1, 0.1 μg/mL, and control; compound final concentration: between 10 pg/mL and 100 μg/mL, and control) and incubated for 72 h at 37 °C in a humidified atmosphere containing 5% CO_2_. After cultivation, 10 μL of 3-(4,5)-dimethyl-2-thiazoyl-2,5-diphenyl-2H-tetrazolium bromide (MTT reagent) solution was added to the microtiter wells. After incubation for 4 h at 37 °C, 100 μL of isopropanol was added to solubilize the MTT-formazan product. The absorbance at 450 nm was measured with a microplate reader.

### 3.7. Apoptosis Analysis

Detection of apoptosis was performed using The Muse Annexin V & Dead Cell Assay Kit (Merck, Darmstadt, Germany) according to the manufacturer’s protocol. MT-1 and MT-2 cells incubated in the presence or absence of compound **3** and **26** for 72 h were collected by centrifugation (310× *g* at 4 °C for 10 min), suspended in 100 μL of RPMI 1640 medium, and incubated with 100 μL of Annexin V reagent at room temperature for 20 min. Cells were measured by a Muse Cell Analyzer (Merck, Darmstadt, Germany).

## 4. Conclusions

In conclusion, as part of our investigations of Cupressaceae plants, extracts of *T. occidentalis* showed potent inhibitory effects against MT-1 and MT-2 cell lines. We isolated 32 compounds (eight lignans, 18 terpenoids, and six flavonoids) from the extract and examined their structure–activity relationships. Almost all of the isolated lignans inhibited the growth of both tumor cell lines. Several diterpene compounds had moderate activity, of which some have been previously reported to exhibit cytotoxicity in cancer cells. Moreover, several flavonoids showed moderate activity; some of these biflavones have been reported to show anti-cancer properties in some cancers and may induce cell cycle arrest, and inhibitory activities against amyloid-β peptide 42 cytotoxicity in PC-12 cells [45]. We continue to search the biflavone and components of other Cupressaceae plants. We also demonstrated that deoxypodophyllotoxin (**3**) and isoginkgetin (**26**) enhanced apoptosis. Therefore, not only lignans but also diterpenes and/or biflavones may be promising candidates for the treatment of ATL.

## Figures and Tables

**Table 1:** Anti-proliferative activities of the tested plant extracts.

Family	Scientific Name	Parts	EC_50_ (µg/mL)	
			MT-1	MT-2
Cupressaceae	*Biota orientalis*	Leaves	>100	>100
		Stems	>100	>100
	*Juniperus chinensis* var. *kaizuka* Hort	Leaves	43.4	61.1
		Stems	18.1	10.8
	*Juniperus rigida*	Leaves	14.0	13.2
		Stems	>100	>100
	*Thuja occidentalis*	Leaves	1.65	1.38
		Stems	5.51	4.21
		Cones	1.74	0.50
	*Thujopsis dolabrata*	Aerial parts	2.40	0.76

The presented data are the mean of three independent experiments.

**Table 2:** Anti-proliferative activity of compounds **1**–**32**, doxorubicin, and etoposide standards (EC_50_).

Compound	EC_50_ (µM)	
	MT-1	MT-2
**1**	0.115	0.134
**2**	0.970	1.3
**3**	0.0058	0.0033
**4**	0.20	0.12
**5**	43.7	16.5
**6**	0.750	0.675
**7**	9.46	7.98
**8**	0.611	0.175
**9**	>476	>476
**10**	109	118
**11**	63.9	125
**12**	174	200
**13**	>314	>314
**14**	121	64.9
**15**	>301	172
**16**	142	133
**17**	>301	>301
**18**	109.5	30.49
**19**	25.8	19.2
**20**	17.7	22.3
**21**	103	125
**22**	119	30.0
**23**	74.6	140.9
**24**	228	109
**25**	88.1	135.8
**26**	5.26	2.45
**27**	7.07	5.78
**28**	>172	>172
**29**	>172	>172
**30**	8.97	9.78
**31**	>234	>234
**32**	>169	>169
doxorubicin	0.015	0.013
etoposide	0.051	0.065

## Supplementary Materials

The following are available online, contain ^13^C-NMR data of compounds (Table S1: ^13^C-NMR spectroscopic (CDCl_3_) of compounds **1**–**8**. Table S2: ^13^C-NMR spectroscopic (CDCl_3_) of compounds **9**–**11**. Table S3: ^13^C-NMR spectroscopic (CDCl_3_) of compounds **12**–**24**. Table S4: ^13^C-NMR spectroscopic (DMSO-*d_6_*) of compounds **25**–**30**. Table S5: ^13^C-NMR spectroscopic (CDCl_3_) of compounds **31** and **32**.)

## Sample Availability

Samples of the compounds are not available from the authors.
