# Supplementary material for: Screening of Promising Chemotherapeutic Candidates from Plants against Human Adult T-Cell Leukemia/Lymphoma (VII): Active Principles from Thuja occidentalis L
Source: Molecules. 2021 Dec 15;26(24):7619. doi: 10.3390/molecules26247619 (PMC8707666; doi:10.3390/molecules26247619)
Supplement: Supplementary file 1 [file molecules-26-07619-s001.zip › molecules-1492229-supplementary.pdf]

Contents

Table S1. ^{13}C-NMR spectroscopic ($CDCl_3$) of compounds **1–8**.

Table S2. ^{13}C-NMR spectroscopic ($CDCl_3$) of compounds **9–11**.

Table S3. ^{13}C-NMR spectroscopic ($CDCl_3$) of compounds **12–24**.

Table S4. ^{13}C-NMR spectroscopic (DMSO-d_6) of compounds **25–30**.

Table S5. ^{13}C-NMR spectroscopic ($CDCl_3$) of compounds **31** and **32**.

Table S1. ^{13}C-NMR spectroscopic ($CDCl_3$) of compounds **1–8**.

Compound	**1**	**2**	**3**	**4**	**5**	**6**	**7**	**8**
1	133.1	132.1	128.3	128.3	127.3	131.6	131.4	131.1
2	106.3	108.9	108.5	108.8	109.5	108.4	108.6	108.6
3	147.9	148.6	147.1	146.9	153.8	148	147.9	148
4	147.8	147.6	146.8	146.8	148.5	146.5	146.4	146.5
5	109.9	110.6	110.5	109.9	106.1	108.8	108.0	108.1
6	131.3	131.9	130.7	130.5	137.5	121.6	121.5	121.7
7	72.9	66.8	33.1	32.1	193.4	35.2	39.4	39.3
8	40.9	38.8	32.8	33.0	43.4	41.0	36.5	37.6
9	71.3	67.5	72.0	72.7	70.5	71.1	72.9	72.2
1'	135.3	137.5	136.2	138.2	139.6	133.3	136.6	133
2'	108.6	108.5	108.5	105.2	104.9	106.5	102.2	102.5
3'	152.7	152.7	152.6	153.5	153.8	153.4	153.5	153.6
4'	137.5	135.0	137.3	137.1	138	137.2	137.4	137.9
5'	152.7	152.7	152.6	153.5	153.8	153.4	153.5	153.6
6'	108.6	108.5	108.5	105.2	104.9	106.5	102.2	102.5
7'	44.2	40.5	43.8	45.3	43.5	38.4	72.3	73.9
8'	45.4	44.0	47.5	46.4	46.7	46.5	52.7	50.9
9'	174.2	174.9	174.8	178.3	175.5	178.4	178.4	176
-O-CH2-O-	101.5	101.6	101.2	101.1	102.2	101.1	101.2	101.3
3'-OMe	56.4	56.3	56.3	56.3	56.3	56.2	56.2	56.2
4'-OMe	60.7	60.7	60.7	60.8	60.8	60.9	61.0	60.9
5'-OMe	56.4	56.3	56.3	56.3	56.3	56.2	56.2	56.2
-Me-COO-								169.2
								21.0

Table S2. ^{13}C-NMR spectroscopic ($CDCl_3$) of compounds **9–11**.

Compound	**9**	**10**	**11**
1	49.5	79.9	59.4
2	25.3	26.8	123.1
3	28.7	39.5	140.3
4	211.3	71	39.9
5	57.1	46.3	25.1
6	55.8	23.1	36.7
7	46.8	141.9	32.7
8	23.1	116.1	37.4
9	42.1	40.7	24.5
10	73	37.7	37.4
11	29.5	35	32.8
12	15.6	21.8	37.3
13	21.9	21.2	24.8
14	20.4	11.7	39.4
15	29.5	29.8	28
16			22.7
17			22.6
18			19.7
19			19.8
20			16.2

Table S3. ^{13}C-NMR spectroscopic ($CDCl_3$) of compounds **12–24**.

Compound	**12**	**13**	**14**	**15**	**16**	**17**	**18**	**19**	**20**	**21**	**22**	**23**	**24**
1	38.2	33.4	38.9	34.5	36.2	36.3	38.5	38.4	37.6	38.9	39.6	38.4	39.2
2	18.2	17.9	18	18.2	18.0	17.9	18.7	18.7	18.3	19.2	19.5	20.1	20.0
3	36.8	36.1	37.1	36	33.7	37.0	35.1	34.7	34.9	41.7	41.6	39.6	38.1
4	46.7	46.4	46.3	47	46.4	46.8	37.8	37.3	37.8	37.5	33.3	44.2	44.2
5	41.8	44.6	45.1	41.2	44.7	44.8	43.9	37.6	42.6	50.3	49.6	56.4	56.4
6	31.6	33.6	25.2	25	33.6	34.8	18.8	27.7	36.0	19.3	19.4	23.5	26.1
7	73.3	199.7	121	78.2	199.5	198.8	30.1	68.8	199.7	29.8	28.8	40.4	38.8
8	138.4	129.1	135.7	125.3	129.3	129.2	134.8	136.1	130.8	127.3	134.0	148.4	148.1
9	46.1	166.0	52.1	141.7	165.8	164.5	147.4	147.5	153.7	148.7	143.2	56.7	56.5
10	38.0	39.2	35.1	37.7	39.3	39.2	37.3	37.9	37.6	33.3	37.7	38.7	40.7
11	18.1	22.9	20.1	21.0	23.0	21.6	123.7	126.7	123.7	111.0	123.0	26.1	18.0
12	34.2	34.7	36.1	35.3	34.8	34.7	124.2	124.9	132.6	150.7	114.3	134.1	41.4
13	37.4	34.4	36.8	34.9	34.5	34.9	145.5	146.5	146.8	131.4	152.0	133.6	73.7
14	134.8	36.8	46.1	39.1	37.0	36.8	126.8	127.7	125.0	126.6	131.0	141.8	145.1
15	148.0	145.1	150.3	146.0	145.2	145.1	33.4	33.5	33.7	26.8	27.1	109.8	111.6
16	110.9	111.9	109.3	111.4	120.0	111.9	23.9	23.9	23.9	22.7	20.3	12.8	28.1
17	25.7	28.0	21.5	27.4	28.1	28.0	23.9	24.0	24.0	22.6	20.3	107.5	106.6
18	182.6	182.3	182.6	183.3	181.4	177.8	71.8	71.1	71.1	33.4	33.2	29.1	29.0
19	16.8	16.3	17.2	16.5	16.4	16.4	17.3	17.7	17.3	21.6	21.6	180.4	182.4
20	14.5	18.2	15.3	18.4	18.3	18.2	25.2	24.1	23.8	24.8	25.2	11.8	12.7
-COOMe						52.0							
-OMe				56.1									

Table S4. ^{13}C-NMR spectroscopic (DMSO-d_6) of compounds **25–30**.

Compound	**25**	**26**	**27**	**28**	**29**	**30**
2	163.7	163.2	163.5	163.7	163.9	163.6
3	103.5	103.6	103.5	104.8	104.4	103.6
4	182.1	181.6	181.9	182.7	181.8	181.6
5	160.5	161.4	161.5	160.5	162.4	161.1
6	98.8	98.8	98.5	98.4	98.0	98.9
7	164.1	162.8	165.1	165.6	165.0	163.1
8	98.8	94.1	92.6	92.6	92.6	93.9
9	157.4	157.4	157.3	157.7	157.2	157.3
10	103.7	103.6	103.6	105.5	104.6	103.7
1'	121.0	122.5	122.3	123.3	120.3	123.8
2'	131.4	128.0	128.2	128.3	128.3	128.1
3'	121.4	121.8	121.7	123.3	122.3	115.3
4'	161.0	160.5	160.6	159.7	160.3	161.4
5'	116.2	111.6	111.7	111.9	111.7	115.3
6'	127.8	130.8	130.7	131.3	130.7	128.1
7-OMe				56.2	56.3	
4'-OMe				55.5	55.8	
2"	163.8	164.5	163.6	163.7	163.4	164.4
3"	102.6	103.3	102.5	104.0	103.7	102.3
4"	181.7	181.9	182.0	182.2	182.0	181.6
5"	159.5	160.4	160.4	162.6	162.4	152.8
6"	94.0	98.9	98.6	99.8	95.3	125.2
7"	161.5	161.4	161.7	162.1	161.3	154.0
8"	103.7	103.6	103.8	103.2	102.7	94.9
9"	154.5	154.3	154.3	154.6	153.3	154.0
10"	104.0	103.6	103.5	103.2	103.9	103.7
1'''	120.0	122.8	121.2	122.1	121.7	121.2
2'''	128.2	127.7	128.0	127.7	128.0	128.3
3'''	115.8	114.4	115.8	114.5	115.8	115.9
4'''	161.9	162.1	161.0	162.6	161.0	160.8
5'''	115.8	114.4	115.8	114.5	115.8	115.9
6'''	128.2	127.7	128.0	127.7	128.0	
4"-OMe				55.8		

Table S5. ^{13}C-NMR spectroscopic ($CDCl_3$) of compounds **31** and **32**.

Compound	**31**	**32**
1	37.3	37.5
2	31.7	30.3
3	71.8	78.6
4	42.4	39.4
5	140.8	141.4
6	121.7	122.0
7	31.9	32.2
8	32.0	32.1
9	50.2	50.4
10	36.6	37.0
11	21.1	21.3
12	39.8	40.0
13	42.4	42.5
14	56.8	56.9
15	24.3	24.5
16	28.3	28.6
17	56.1	56.3
18	11.9	12.0
19	19.4	19.1
20	36.2	36.4
21	18.8	19.5
22	34.0	34.2
23	26.2	26.4
24	45.9	46.1
25	29.3	29.5
26	19.1	19.3
27	19.8	20.0
28	23.1	23.4
29	12.0	12.2
1'		102.6
2'		78.1
3'		75.4
4'		71.7
5'		78.5
6'		62.9
